# Factors Possibly Associated with Mortality in Intubated COVID-19 Patients: A Retrospective Study

**DOI:** 10.3390/pathogens11020235

**Published:** 2022-02-11

**Authors:** Lilia Esther Ramírez-Plascencia, Ana Paulina Vázquez-León, Almendra Villaseñor-Magaña, Marisela Correa-Valdéz, Sandra Carrillo-Ibarra, Sonia Sifuentes-Franco

**Affiliations:** 1 Anaesthesiology Service, Civil Hospital of Guadalajara Dr. Juan I. Menchaca, Guadalajara 44340, Mexico; buffy-sommers@hotmail.com (L.E.R.-P.); paulina_0813@hotmail.com (A.P.V.-L.); dra.almendravillasenorm@gmail.com (A.V.-M.); marisela.correa@hotmail.com (M.C.-V.); 2 University Center for Health Sciences, University of Guadalajara, Guadalajara 44340, Mexico; 3 School of Health Sciences, Universidad del Valle de México, Zapopan 45010, Mexico; sandra_carrillo@my.uvm.edu.mx; 4 Laboratory of Biological Systems, Department of Health Sciences, Valles Campus (CUValles), University of Guadalajara (UDG), Ameca 46600, Mexico; 5 Department of Health Sciences—Disease as an Individual Process, Tonalá Campus, University of Guadalajara (UDG), Tonalá 48525, Mexico

**Keywords:** COVID-19, intubation, mortality, risk factors, comorbidities

## Abstract

In Mexico, there is a high mortality rate among patients intubated because of COVID-19. The objective of this study was to investigate the associations of age, comorbidities, and biochemical parameters with the in-hospital mortality of COVID-19 patients. A retrospective study of 79 intubated patients admitted to one hospital in Jalisco, Mexico, between July 2020 and January 2021 was performed. Demographic and clinical characteristics were collected. The mean age was 57.7 (±12.8) years, with 83.5% non-survivors and 16.5% survivors. Age, lactate dehydrogenase (LDH) and D-dimer levels were found to be significantly higher in the non-survivor group (*p* = 0.011, *p* = 0.026, *p* = 0.007, respectively). Patients ≥58 years had a high risk of mortality (OR = 7.017). Significant correlations were also found in some of the study variables: LDH levels and leukocyte count (r = 0.388, *p* = 0.034) and CRP levels and fibrinogen (r = 0.692, *p* ˂ 0.001) in the patients ˂58 years. Leukocyte count with LDH levels (r = 0.381, *p* = 0.024) were significant in the group ≥58 years. No significant difference was observed in the presence of diabetes mellitus (DM) and hypertension. In conclusion, according to logistic regression analysis, age over 58 years represents the main factor associated with mortality in these patients.

## 1. Introduction

After the first cases of a previously unknown disease manifesting as a respiratory tract infection emerged in Wuhan, China in late 2019 [1], on 10 April 2020, the World Health Organization (WHO) declared the coronavirus disease 2019 (COVID-19) as a pandemic because the number of cases had increased to more than 1.7 million, confirmed in more than 210 countries [2]. To date, more than 270.7 million cases have been reported globally, with approximately 5.3 million deaths [3]. However, it is important to note that the figures are constantly changing. According to some reports, the mortality associated with COVID-19 has decreased with timely vaccination [4].

The clinical manifestations of COVID-19 are usually in the respiratory tract; it presents symptoms that range from mild to severe or fatal. It manifests as a cold but can be aggravated to a viral-type pneumonia and acute respiratory distress syndrome [5]. Approximately 14–30% of patients hospitalized for COVID-19 develop respiratory failure, and 5% require intubation. The mortality rate is high in patients who develop severe disease; 49–67% of the patients are admitted to the intensive care unit, and more than 50% require intubation or mechanical ventilation [6,7]. According to a multicentre study conducted in Wuhan, approximately 97% of patients requiring invasive mechanical ventilation die. To date, it is known that the presence of comorbidities such as diabetes mellitus (DM), systemic arterial hypertension (SAH), and cancer are associated with the mortality rate, in addition to high concentrations of D-dimer, C-reactive protein, and low lymphocyte counts in those patients with severe disease who undergo intubation [8,9]. Therefore, the intubation process has been one of the greatest challenges in patients with COVID-19.

When the intubation process is performed, the intensity of the laryngeal stimulus is related to the hemodynamic response and the release of catecholamines. These processes can worsen when there is not an adequate depth of anaesthesia and neuromuscular relaxation. In addition, the process is complicated when laryngoscopy is performed in a patient with a difficult airway [10].

In patients with COVID-19, the intubation process is complicated due to minimal or no respiratory reserve, depletion of compensatory mechanisms, and personal protective equipment hindering the performance of the procedure. Additionally, strict infection risk control restricts available supplies and support personnel. As a result, careful airway assessment is often not possible [6].

Thus, the present study aimed to investigate the associations of age, comorbidities, and biochemical parameters with the in-hospital mortality of patients with COVID-19.

## 2. Materials and Methods

### 2.1. Patients and Study Design

The study was carried out through a retrospective design at the O.P.D. Hospital Civil De Guadalajara “Dr. Juan I. Menchaca” (urban public hospital) in Guadalajara, Mexico. The study was based on the collection of data obtained during routine clinical practice in patients older than 18 years who required hospitalization and intubation, with confirmatory SARS-CoV-2 infection by polymerase chain reaction (PCR) test between July 2020 and January 2021. The study received an exemption from our institutional review committees as only existing data in medical records were analysed. The identity of the patients remained anonymous to the researchers.

### 2.2. Data Collection

The following variables were collected: age, gender, comorbidities, vital signs prior to the intubation process (including blood pressure, respiratory rate, heart rate, and oxygen saturation), and data on inflammatory markers including white blood cell, C-reactive protein (CRP) levels, D-dimer, lactate dehydrogenase (LDH), fibrinogen, partial thromboplastin time (PTT) and prothrombin time (PT). Likewise, the way in which the intubation was performed was identified, either by direct laryngoscope or video. To ensure the validity of the included cases, the analysis was carried out by three researchers. Likewise, the following were excluded: patients whose medical records showed the confirmatory PCR test for COVID-19 was negative and patients under 18 years of age.

### 2.3. Statistical Analysis

The data are shown as descriptive statistics. For quantitative variables, the mean and standard deviation (SD) were used, and for qualitative variables, frequency and percentage were used. To evaluate the association of the variables, Mann–Whitney U and χ2 tests were used as appropriate. Pearson’s test was used for correlations. Both R and SPSS version 20.0 Chicago II were used for all analyses. All *p* values ≤0.05 were considered significant.

## 3. Results

**Figure 1:**
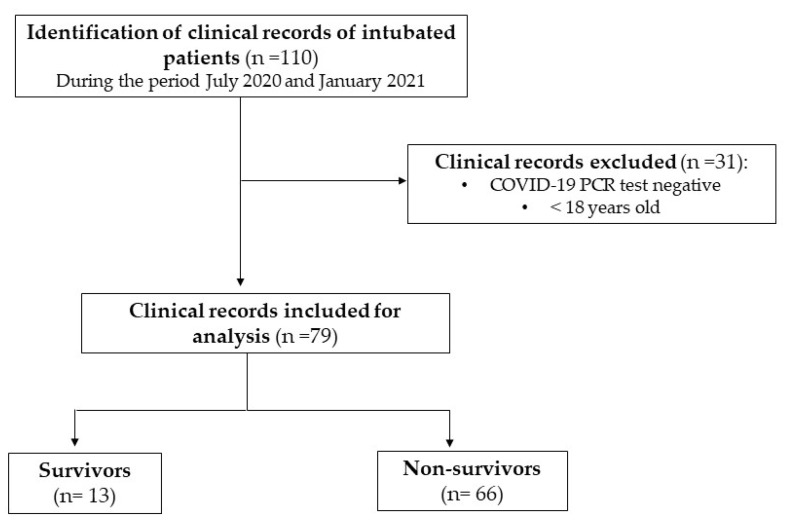
Study population.

One hundred and ten clinical records of patients who required intubation due to hypoxia were identified in the public O.P.D. Hospital Civil de Guadalajara “Dr. Juan I. Menchaca, between July 2020 and January 2021. Of these, only 79 clinical records met the criteria for inclusion in the analysis (Figure 1). It was found that the average age was 57.7 ± 12.8 years; 73.4% corresponded to men, and the remaining 26.6% were women. Most of the patients at the time of intubation already had comorbidities in addition to COVID-19. The most common comorbidity was SAH (46.8%), followed by type 2 diabetes mellitus (DM2) (44.3%) and chronic kidney disease (3.8%), as well as other diseases, such as lung cancer, breast cancer, hypothyroidism, and liver cirrhosis (1.26%). Overall, 27.8% of the patients were smokers, and the average weight was 86.6 ± 23.9 kg (Table 1).

### Clinical Characteristics and Mortality

The overall mortality rate was 83.5%. As shown in Table 2, the study population was divided between patients who survived and non-survivors. Different parameters were compared to determine if any were associated with mortality. In the group of non-survivors, the average age was 59.5 ± 12.0, significantly higher than the survivors’ group (*p* = 0.011), and a statistically significant laboratory value associated with mortality was elevated LDH (*p* = 0.026). 

Another characteristic that showed a difference between the distribution was the presence of obesity and its categories (*p* = 0.03). Likewise, it was observed that the oxygen saturation was lower in the non-survivors group. However, it was not statistically significant.

No significant difference was found in the frequency of the blood types between the two groups (*p* = 0.591). Similarly, the same was observed for gender and the presence of DM2 and SAH (*p* = 0.473; *p* = 0.519 and *p* = 0.167, respectively) (Table 2).

The odds ratio was calculated for the age over 58 years; the value obtained was 7.017, *p* = 0.007, and the confidence interval was 1.44–34.02. 

As shown in Table 3, the laboratory parameters were correlated in the group of non-survivors. In said correlations, age (greater than 58 years) was maintained as a control variable. In the group ˂58 years old, CPR and fibrinogen levels were positively correlated (r = 0.692, *p* ˂ 0.001). Likewise, the leukocyte count and LDH levels showed a significant correlation (r = 0.388, *p* = 0.034). On the other hand, in the group ≥58 years, statistical significance was observed between the correlations of fibrinogen and D-dimer. This correlation was negative (r = −0.357, *p* = 0.035). In the case of the correlation between BMI and LDH levels, as well as the leukocyte count with LDH levels, a positive correlation was observed (r = 0.322, *p* = 0.05; r = 0.381, *p* = 0.024, respectively).

Different parameters were revealed to possibly be associated with mortality in patients who required intubation due to COVID-19. These were age, D-dimer, and LDH levels, as well as the presence of obesity. Therefore, to assess the effects of the set of variables studied, a bivariate logistic regression analysis was performed. This revealed that age over 58 years and high levels of D-dimer and LDH could be explanatory variables of mortality. However, after adjusting for these variables in the logistic regression analysis, only age over 58 years was found to be significant (Table 4).

## 4. Discussion

The present study investigated different variables and parameters that could be associated with mortality in 79 patients with COVID-19 who underwent intubation at a hospital in Guadalajara, Mexico. This study demonstrated that age is an important predictor; that is, patients older than 58 years of age had a higher risk of death (7.07), which is consistent with other reports in which advanced age has already been shown to be associated with increased risk in the mortality of patients with COVID-19, independent of the intubation procedure [11,12]. Likewise, this study showed that the presence of comorbidities such as obesity also represents a risk factor for mortality in these patients. This supports previous information, where it was shown that even in young patients who presented with obesity, it increased mortality [13,14]. These results indicate that obesity can exacerbate the clinical manifestation of COVID-19, as well as be an important risk factor for mortality in these patients, possibly through the following processes. The inflammatory microenvironment of adipose tissue during obesity is generated by an imbalance between anti-inflammatory and pro-inflammatory cytokines, decreasing the secretion of anti-inflammatory cytokines and increasing pro-inflammatory cytokines such as tumour necrosis factor α (TNF-α), IL-1β, interferon γ (IFN-γ) and IL-6, which leads the development of a chronic inflammatory disease [15]. Likewise, in obese patients, the limitation of thoracic and expansion movements hinders respiratory function, generating an inadequate ventilatory function in patients with COVID-19 [16].

Although it was shown in previous studies that comorbidities such as DM2 and SAH play a role in increasing the risk of mortality in patients with COVID-19, this effect was not observed in this study. However, it is important to mention that the different comorbidities related to metabolic disorders, such as DM2 and SAH, increase the susceptibility to develop severe COVID-19. Although no association was observed between the frequency of diabetic and hypertensive patients with mortality, we consider that it could be related to the lack of timely diagnosis of these diseases in countries such as Mexico [17]. That is why we consider it important to develop new studies with larger sample sizes. An important finding that we found in this study is the biomarker LDH. We observed that, at high values, it can be a factor associated with mortality in patients undergoing intubation. This can be explained by the deterioration of cardiac function caused by ageing and by previous pathologies, such as hypertension and heart disease (in some cases). However, it is important not to rule out that it could be an indicator of the COVID-19 damage process because it has previously been shown that in severe cases, there is multi-organ damage. 

In addition, it has been previously reported that elevated LDH values are an independent risk factor for COVID-19 [18]. Hypoxia and inflammatory processes associated with the disease lead to elevated LDH as well as an increase in the number of white blood cells to mediate inflammatory processes. Findings related to the participation of this biomarker in processes with an inflammatory component could be explained by the positive correlation between BMI and LDH levels found in this study. It is evident that in processes such as obesity, the inflammatory response plays a fundamental role [15], as well as in ageing, since in our study, this behaviour was only observed in the group of patients ≥58 years old.

Regarding leukocytes, it has already been reported that those patients diagnosed with COVID-19 who present with higher levels of leukocytes have a higher risk of death [19]. In our study, there were no significant differences regarding the level of leukocytes in either group. However, when correlating leukocyte and LDH levels in the group of non-survivors, we found a positive correlation regardless of age. The increase in leukocytes is accompanied by an increase in LDH levels; the elevation of both factors is related to the inflammatory process and damage generated by the severity of the disease.

D-dimer is a biomarker strongly correlated with diseases associated with haemostasis, such as venous thromboembolism (VTE) [20]. Likewise, a large number of studies have shown that the levels of D-dimer are significantly higher in patients with COVID-19 who do not survive or who have greater severity than in patients with mild COVID-19. This reflects the state of hypercoagulability already described by other authors [12,21]. Our study agrees that D-dimer is a biomarker associated with mortality in patients with COVID-19. However, correlation results segmented by age ≥58 years showed a negative association between fibrinogen levels and D-dimer, but in patients with COVID-19, some laboratory abnormalities and complications for thrombosis may occur; among these are prolonged prothrombin time (PT), increased fibrinogen, increased platelet count, and increased D-dimer [22]. In this study, we observed something similar without adjusting for age. However, when adjusting for patients older than 58 years, this correlation was not consistent with the above, which could be due to the high variability of the data and the sample size.

Our results show a significant correlation between the age of the patients and D-dimer levels, consistent with previous reports [23]. The explanation for the increased levels of D-dimer in older adults is not totally clear. An intriguing hypothesis is that the increase in D-dimer levels as age increases is due to a pro-inflammatory and oxidative state, typical of ageing, as well as the presence of comorbidities.

Fibrinogen is a biomarker that has previously been shown to be elevated in patients with severe COVID-19 disease [24]. Tang N. et al. found that fibrinogen levels were not different between surviving and non-surviving COVID-19 patients [25]. This is similar to our results, where fibrinogen seems to be elevated in all patients, without any differences found between the two groups. However, the values of fibrinogen were positively correlated with CRP in the group of non-survivors. These results are similar to those reported by Sui J et al., where patients with values ≥489.0 mg/dL of fibrinogen had high CRP values compared to those with lower fibrinogen values [26]. Therefore, this provides evidence that elevated fibrinogen is associated with inflammatory processes, in addition to coagulopathy, and it may be a biomarker to define the course of the disease.

In general, it is important to mention that the combination of different risk factors, such as obesity, advanced age, and cardiovascular comorbidities as well as alterations in coagulation, should not be lost sight of at the time of clinical practice while also looking for new tools to help the patients survive. It was also observed that the blood group is not associated with mortality, as has been described in previous studies [27]. In the case of the population analysed, the blood group A Rh + was more frequent, followed by O Rh +, which is congruent with the frequency of these blood groups in the Mexican population [28]. 

Previous studies using logistic regression models have shown that advanced age and the presence of age-related diseases can predict mortality in patients hospitalized for COVID-19 [29], in addition to being predictors of early mortality after intubation [11]. Unlike these investigations, comorbidities were not factors associated with mortality in the results of the logistic regression carried out in our investigation, but it was shown that age over 58 years and high levels of D-Dimer and LDH could be explanatory factors of mortality. Once the adjustments to the model were made, it was observed that only advanced age is related to mortality during intubation. This finding can be explained by the decrease in respiratory function and immune response related to age.

## 5. Study Limitations

There are some limitations to our study. Firstly, the study focused on the retrospective analysis of clinical records from a small hospital in the city of Guadalajara, Mexico. Likewise, the sample size was limited to patients admitted to this centre who required intubation. However, it supports clinical evidence about the knowledge of prognostic markers in specific populations. This helps to identify patients at high risk of mortality and in the search for therapeutic tools that reduce their risk.

## 6. Conclusions

According to the results of this study, the main co-morbidities in patients intubated due to COVID-19 were hypertension, diabetes, and obesity. Age was associated with mortality. Patients in the non-survivor group had a higher age range and a higher degree of obesity. Correlations were presented between proinflammatory clinical variables (leukocytes, LDH, D-dimer) in the group aged ≥58 years, and the results of the logistic regression analysis showed that age ≥58 years is one of the main factors associated with mortality in patients intubated due to COVID-19.

## Figures and Tables

**Table 1:** Demographic and clinical characteristics of all patients undergoing intubation.

Age, Years	57.7 ± 12.8
Gender, n (%)	
Male	58 (73.4)
Female	21 (26.6)
Comorbidities, n (%)	
SAH	37 (46.8)
DM2	35 (44.3)
Prediabetes	2 (2.5)
CKD	3 (3.8)
Smoker, n (%)	22 (27.8)
SBP, mm Hg	134.5 ± 25.9
DBP, mm Hg	76.5 ± 14.0
HR, bpm	101.7 ± 19.9
RR, bpm	33.2 ± 8.6
SpO2, %	71.03 ± 16.1
Weight, kg	86.6 ± 23.9

SAH = Systemic arterial hypertension; DM2 = Type 2 diabetes mellitus; CKD = Chronic kidney disease; SBP = Systolic blood pressure; DBP = Diastolic blood pressure; HR = Heart Rate; RR = Respiratory rate. SpO2 = Oxygen saturation. Mean ± SD and frequency (percentage).

**Table 2:** Clinical characteristics and their significance in mortality.

	Survivors (n = 13)	Non-Survivors (n = 66)	*p*
Age, years	48.5 ± 13.5	59.5 ± 12.0	0.011 *
Gender, n (%)			0.473
Male	9 (69.2)	49 (74.2)	
Female	4 (30.8)	17 (25.8)	
Blood group, n (%)			0.591
A	9 (69.2)	39 (59.1)	
O	4 (30.8)	25 (37.9)	
AB	0 (0)	2 (3.0)	
SBP, mm Hg	135.6 ± 22.3	134.2 ± 26.7	0.856
DBP, mm Hg	80.2 ± 10.9	75.5 ± 14.2	0.296
HR, bpm	96.9 ± 16.3	102.7 ± 20.5	0.632
BF, bpm	32.6 ± 9.5	33.3 ± 8.5	0.768
SpO2, %	75.8 ± 10.3	70.1 ± 16.9	0.566
Weight, kg	90.9 ± 22.3	85.7 ± 24.3	0.514
CRP, mg/mL	154 ± 107.8	151.9 ± 98.1	0.509
Obesity, n (%)			0.03 *
Overweight	5 (38.4)	7 (10.6)	
Grade 1 obesity	0 (0)	11 (16.7)	
Grade 2 obesity	2 (15.4)	5 (7.5)	
Grade 3 obesity	3 (23.1)	10 (24.2)	
DM2, n (%)	6 (46.1)	28 (42.4)	0.519
SAH, n (%)	4 (30.7)	33 (50.0)	0.167
D-Dimer, ng/mL	469.4 ± 263.2	2039 ± 1750.2	0.007 *
Leukocytes, ×10^3^ cells/µL	14.2 ± 3.8	14.9 ±6.2	0.899
Fibrinogen, mg/dL	907.2 ± 250	807.7 ± 270	0.146
PT, seconds	13.3 ± 1.3	13.8 ± 2.1	0.668
PTT, seconds	31.3 ± 9.0	34.5 ± 13.7	0.582
LDH, U/L	374.4 ± 129	514.0 ±122	0.026 *
INR	1.2 ± 0.12	1.26 ± 0.18	0.586

SAH = Systemic arterial hypertension; DM2 = Type 2 diabetes mellitus; CKD = Chronic kidney disease; SBP = Systolic blood pressure; DBP = Diastolic blood pressure; HR = Heart Rate; RR = Respiratory rate; CRP = C Reactive Protein. Mean ± SD and frequency (percentage). *p* = Mann–Whitney U and χ2 tests were used for groups comparison; * *p* ≤ 0.05 values were considered statistically significant.

**Table 3:** Clinical correlations in non-survivor group (adjusted for age).

	Age ˂ 58 Years		Age ≥ 58 Years	
Fibrinogen (mg/dL)				
	r	*p*	r	*p*
CRP (mg/mL)	0.692	˂0.001 *	0.310	0.070
D-Dimer (ng/mL)	−0.270	0.157	−0.357	0.035 *
**LDH (U/L)**				
Leukocytes (×10^3^ cells/µL)	0.388	0.034 *	0.381	0.024 *
BMI (kg/m^2^)	−0.052	0.784	0.322	0.050 *

CRP = C Reactive Protein; BMI = Body Mass Index. Pearson’s correlation test was used; * *p* ≤ 0.05 values were considered statistically significant.

**Table 4:** Logistic regression of risk factors associated with mortality.

Variable	Logistic Regression		Logistic Regression (Adjusted)	
	OR (95% IC)	*p*	OR (95% IC)	*p*
Age ≥58 years	16.4 (1.14–235.58)	0.039 *	10.83 (1.22–95.79)	0.032 *
SAH	1.6 (0.221–11.55)	0.642	---	---
DM2	1.4 (0.207–8.98)	0.748	---	---
High D-dimer levels	6.4 (1.01–42.72)	0.050 *	3.4 (0.73–15.65)	0.199
High CRP levels	0.25 (0.038–1.63)	0.148	---	---
High LDH levels	9.2 (1.055–79.56)	0.045 *	4.13 (0.744–22.96)	0.105
High Fibrinogen	5.4 (0.35–83.52)	0.230	---	---
High Leukocytes	0.66 (0.086–5.06)	0.689	---	---
BMI > 25 kg/m^2^	0.129 (0.015–1.08)	0.059	---	---

SAH = Systemic arterial hypertension; DM2 = Type 2 diabetes mellitus; CRP = C Reactive Protein; LDH = Lactate dehydrogenase; BMI = Body Mass Index. * *p* ≤ 0.05 values were considered statistically significant. --- indicates that in the adjusted logistic regression these variables are not included in the analysis.
